# Mucopolysaccharidoses—What Clinicians Need to Know: A Clinical, Biochemical, and Molecular Overview

**DOI:** 10.3390/biom15101448

**Published:** 2025-10-12

**Authors:** Patryk Lipiński, Agnieszka Różdżyńska-Świątkowska, Karolina Wiśniewska, Joanna Rusecka, Agnieszka Ługowska, Zbigniew Żuber, Aleksandra Jezela-Stanek, Zuzanna Cyske, Lidia Gaffke, Karolina Pierzynowska, Grzegorz Węgrzyn, Anna Tylki-Szymańska

**Affiliations:** 1 Institute of Clinical Sciences, Maria-Skłodowska-Curie Medical Academy, 00-136 Warsaw, Poland; joanna.rusecka@uczelniamedyczna.com.pl; 2 Department of Pediatrics, Bielanski Hospital, 01-809 Warsaw, Poland; 3 Anthropology Laboratory, Children’s Memorial Health Institute, 04-730 Warsaw, Poland; a.rozdzynska-swiatkowska@ipczd.pl; 4 Department of Molecular Biology, Faculty of Biology, University of Gdansk, 80-416 Gdansk, Poland; zuzanna.cyske@ug.edu.pl (Z.C.); lidia.gaffke@ug.edu.pl (L.G.); karolina.pierzynowska@ug.edu.pl (K.P.); grzegorz.wegrzyn@ug.edu.pl (G.W.); 5 MEDGEN Medical Centre, 02-954 Warsaw, Poland; 6 Department of Genetics, Institute of Psychiatry and Neurology, 02-957 Warsaw, Poland; alugipin@yahoo.com; 7 Department of Pediatrics, Faculty of Medicine and Health Sciences, Andrzej Frycz Modrzewski Krakow University, 30-705 Kraków, Poland; zbyszekzuber@interia.pl; 8 Department of Genetics and Clinical Immunology, National Institute of Tuberculosis and Lung Diseases, 01-138 Warsaw, Poland; jezela@gmail.com; 9 Department of Pediatric Nutrition and Metabolic Diseases, The Children’s Memorial Health Institute, 04-730, Warsaw, Poland; atylki@op.pl

**Keywords:** mucopolysaccharidoses, glycosaminoglycans, lysosomes, diagnostic algorithm, biochemical diagnostics, molecular analyses, enzyme replacement therapy, hematopoietic stem cell transplantation, gene therapy

## Abstract

The classification of mucopolysaccharidoses (MPSs) includes the classical types (I; II; III with subtypes A, B, C, and D; IV with subtypes A and B; VI; VII; IX; X), associated with impaired lysosomal degradation of mucopolysaccharides, also known as glycosaminoglycans (GAGs), as a result of deficiency in the specific enzymes responsible for GAG degradation (MPS IIIE has so far been identified only in animal models) and MPS-plus syndrome (MPSPS), which is characterized by an accumulation of undegraded GAGs, arising from impaired endosomal trafficking and inefficient delivery of these compounds to lysosomes (due to the VPS33A protein deficiency with normal GAG-degrading enzyme activities assessed in vitro). The aim of this comprehensive review is to provide physicians with a clinical, biochemical, and molecular overview of MPS manifestation. A brief summary of available and emerging therapies is also presented.

## 1. Background

Mucopolysaccharidoses (MPSs) are a group of diseases associated with impaired lysosomal degradation of mucopolysaccharides (also known as glycosaminoglycans, GAGs), which include five types of disaccharides: heparan sulfate (HS), dermatan sulfate (DS), keratan sulfate (KS), hyaluronan, and chondroitin-6-sulfate (C6S) [1]. GAGs are the main components of the extracellular matrix, and depending on the impaired enzyme type (Table 1), the accumulated GAGs differ, leading to specific abnormalities [2,3,4,5].

Until recently, distinct types of MPS (I, II, III (with subtypes A, B, C, and D), IV (with subtypes A and B), VI, VII, IX) were distinguished, resulting from 11 enzyme deficiencies, have been described [1]. The spectrum of MPSs has now expanded to include MPS X, caused by biallelic pathogenic variants in the *ARSK* (arylsulfatase K) gene, and a putative MPS IIIE subtype associated with *ARSG* (arylsulfatase G) deficiency, although the latter has so far been identified only in animal models and remains unconfirmed in humans [6,7]. In 2017, a new disease described as Mucopolysaccharidosis-Plus Syndrome (MPS-PS) was included in the Online Mendelian Inheritance in Man^®^ (OMIM^®^) database [8,9]. The name of the disease stems from the presence of some clinical and biochemical features (including increased urinary GAG excretion) resembling MPSs and additional signs and symptoms. However, contrary to all other MPS types, MPS-PS is not caused by dysfunction of any lysosomal enzyme but rather by pathogenic variants of the *VPS33A* gene, when present in a homozygous configuration [9]. The product of this gene is involved in endosomal maturation and trafficking. The classification of MPSs is based both on the type of stored GAG and on the specific defective enzyme responsible for its degradation. Enzymatic activity assays allow differentiation between classical MPSs and MPS-PS. Morphological assessment of lysosomes also reveals clear differences: in classical MPSs, these organelles are markedly enlarged due to lysosomal GAG accumulation, whereas in MPS-PS, this phenomenon is not observed, most probably due to stacking of GAGs in endosomes, arising from impaired endosomal trafficking and inefficient delivery of these compounds to lysosomes [10].

MPSs are inherited in an autosomal recessive manner, except for MPS II (Hunter syndrome), which comprises an X-linked recessive disorder. Female carriers are usually asymptomatic; however, they can be exceptionally affected due to abnormalities of the X chromosome, homozygosity, or skewed X inactivation [11].

The genetic causes of MPSs (except MPS-PS) are pathogenic variants in genes encoding enzymes responsible for the breakdown of GAGs. Each type of MPS is caused by the presence of pathogenic variants in a different gene (Table 1) [12]. Furthermore, the same type of MPS, as in the case of MPS III, can be conditioned by defects in different genes, and conversely, pathogenic variants in the same gene can be associated with distinct disorders, as exemplified by MPS IVB and GM1 gangliosidosis, both resulting from *GLB1* defects. The intra-disease variability is mainly due to different underlying molecular defects and the consequent degree of residual enzyme activity. Usually, there is a correlation between the severity of the disease and the age of symptom onset, with more severe disease having an earlier onset. The attenuated forms of MPS are more difficult to diagnose since the disease could progress silently over years and early symptoms could be subtle and may be overlooked by physicians [13].

The aim of this review is to provide physicians with a clinical, biochemical, and molecular overview of MPS manifestation and diagnosis, with a brief summary of available and emerging therapies. It is imperative that clinicians do not miss early opportunities for diagnosis. The time delay between the onset of symptoms and diagnosis of disease has implications for disease management. The phenotypic variability of MPSs makes diagnosis challenging, especially for attenuated forms.

## 2. Biochemical, Molecular, and Cellular Disorders in MPSs

MPSs are progressive, hereditary disorders, and most of them (except MPS-PS; see below for more detailed explanation) arise from the presence of genetic variants causing either a complete absence or significant reduction in the activity of specific lysosomal enzymes responsible for GAG degradation [14]. Undegraded GAGs accumulate in lysosomes, leading to dysfunction of cells, tissues, and eventually entire organs [15]. The activities of these enzymes are closely correlated—each enzyme begins to act only after the previous one has completed its reaction. As a result, the lack of activity of any of them disrupts the entire degradation process [16,17].

However, more recent investigations indicated that MPSs are also associated with the dysfunction of many biochemical and cellular processes that are essential for normal cell function. It is therefore possible that different molecular defects, rather than GAG accumulation alone, play a key role in the pathogenesis of the disease. These pathological changes can be exemplified by dysregulation of apoptosis and autophagy, abnormalities in vesicular transport, defects in proteasomal functions, mitochondrial dysfunctions, disturbances in the cell cycle, and disorders of the cytoskeleton and intracellular signaling [18,19,20,21,22,23,24,25].

Interestingly, dysregulation of the expression of hundreds of genes (between 289 and 893, depending on the MPS type) was reported in cells derived from patients with all tested MPS types, as revealed by transcriptomic analyses [26,27]. Such global changes in the levels of transcripts, and in turn in the gene products (proteins), could explain widespread dysfunctions of organelles and cellular processes. This implies that there are cascades of regulatory disorders, when dysregulation of one gene (coding for a regulator of transcription of other genes) causes further disturbances in the processes of controlling the expression of a battery of different genes. Nevertheless, still, one should ask what the biochemical basis of modification of the expression of the regulatory genes by GAGs is. The solution came from studies on receptors of specific hormones. Namely, it was found that membrane estrogen receptor 1 (GPER1) and oxytocin receptor (OXTR) form aggregates in MPS cells [25,28]. It was then demonstrated that GAGs present in MPS cells can flow out of lysosomes when these organelles are partially damaged due to severe accumulation of the storage material. When present at elevated concentrations, HS and DS were shown to form complexes with GPER1 and OXTR, causing the accumulation of inactive receptors, unable to activate signal transduction pathways [25].

As mentioned above, the mechanism of MPS-PS differs significantly from other MPS types. Contrary to “classical” MPS diseases, in MPS-PS cells, all lysosomal enzymes responsible for GAG degradation are unchanged, while pathological variants occur in a gene coding for the VPS33A protein [29]. Nevertheless, massive GAG accumulation still occurs, which is quite surprising. Only recent studies have indicated what the possible molecular mechanisms of MPS-PS are. First, overacidification of lysosomes was observed, which might result in inefficient GAG degradation despite the presence of all enzymes necessary for this process [30]. Second, the VSP33A protein is a component of two large, multifunctional complexes: the homotypic fusion and protein sorting complex (HOPS) and the class C core endosomal vacuolar binding complex (CORVET). Both of these complexes are involved in the transport of proteins to lysosomes, as well as in autophagy and endocytosis processes [30,31]. Reduced levels of VPS33A protein have been demonstrated in both MPS-PS variants, severe and attenuated, but in both cases, the protein retained its biochemical activity, indicating that the symptoms are caused by a reduced amount of protein due to its excessive degradation in the proteasome, rather than a lack of activity [32,33]. Further studies have indicated that the pathomechanism of MPS-PS may be caused by impaired endosomal transport, which is caused by reduced levels of the VPS33A protein and impaired formation of the HOPS and CORVET complexes. Thus, GAGs cannot be effectively delivered to lysosomes for degradation, resulting in their accumulation, as all enzymes involved in the decay of these carbohydrates are localized in these organelles [10].

In summary, GAG accumulation is the primary biochemical defect in MPSs, caused by the presence of pathogenic variants of genes coding for either proteins involved directly in the degradation of these compounds or a protein required for endosomal trafficking. The pathomechanisms of this group of conditions are more complex and involve the dysregulation of hundreds of genes and dysfunctions of various organelles and cellular processes.

## 3. Clinical Manifestation of MPSs

### 3.1. Main Clinical Symptoms Regarding Accumulated GAGs

When occurring at highly elevated levels, DS has a particularly damaging effect on the formation of connective tissue, while HS has an affinity for and a toxic effect on the central nervous system (CNS).

The accumulation of DS leads to a disturbance in elastogenesis and abnormal bone formation, destructive changes in growth and joint cartilage, synovial membranes, bones, and periarticular soft tissues [34,35]. In the case of MPSs in which DS is stored, namely MPS I, II, VI, and VII, the symptoms of the disease result from an abnormal structure of the connective tissue, which affects many organs and systems (i.e., *dysostosis multiplex*, degeneration of the heart valves, mild hepatomegaly, see Table 2). In MPS I, DS accumulates within the keratocytes, causing them to swell and lose their characteristic morphology, and also in granules throughout all corneal layers. Both GAG deposits cause the disruption of the parallel arrangement of the collagen fibril, leading to corneal clouding. The relatively lower levels of DS in MPS II may contribute to delayed development of corneal clouding or lack thereof. Since the enzymatic defects in MPS I and MPS II both result in the accumulation of HS and DS, the clinical differences between both diseases cannot be explained by GAG species accumulation alone [36].

Abnormal HS levels in the CNS were found to be responsible for dysregulation of neuronal differentiation, growth, and neurotransmission [4,37]. Neuroinflammation, thought to be triggered by HS storage affecting toll-like receptors of microglia, was previously reported for all neurological MPS patients and mouse models. In the CNS, HS occurs as a core component of heparan sulfate proteoglycans (HSPGs). HSPGs are present on the surface of neurons and glial cells, associated with synapses, and integrated into perineuronal nets (PNNs). Their physiological roles include regulation and modulation of cell signaling. HSPGs control axon guidance, neuronal development, and synapse formation and are also found in basement membranes, where they preserve the integrity of the blood–brain barrier (BBB). One of the most important processes regulated by HS and HSPGs is the morphogenesis of developing tissues, along with the maintenance of normal cellular functions and intercellular communication throughout the body. Thus, they are critical for the proper formation of brain structures [4,37]. In the context of neurodegenerative diseases, HS accumulation in the brain has been shown to bind amyloid precursor protein (APP), amyloid-β (Aβ), and tau protein, thereby promoting Aβ and tau aggregation. A recent study demonstrated elevated levels not only of these proteins but also of their aggregates in fibroblasts derived from patients with all MPS III subtypes [38]. That report showed excessive levels of alpha-synuclein and TDP-43 protein as well as their aggregates. Note that accumulation of toxic aggregates and HS accumulation leads to excessive ROS production. CNS cells possess relatively weak antioxidant defenses, making them highly vulnerable to damage. These findings provided a direct link between HS accumulation in MPS III and the typical clinical manifestations, such as behavioral disturbances (ADHD-like symptoms, hyperactivity, or aggressive behavior), loss of learning ability, speech impairments, and other cognitive deficits.

Symptoms of CNS involvement (Table 2) are present in MPS III A-D (Sanfilippo disease), MPS I-H (Hurler disease, severe neuronopathic form), neuronopathic forms of MPS II, and MPS VII. In MPS III, the somatic (visceral) symptoms are heterogeneous and may be less severe than in other forms of MPS (with storage of DS mainly).

Patients with MPS VI accumulate DS only and present with severe *dysostosis multiplex*, joint stiffness, coarse facial features, and corneal clouding, in the absence of impaired intellectual development.

MPS IV predominantly affects tissues rich in keratan sulfate (KS), including cartilage, corneas, and heart valves, explaining the predominant phenotypic features. A comparison of the clinical picture of the individual types of MPS is presented in Table 2.

### 3.2. Growth Dynamics

In MPSs, birth length is consistently normal or above average, with early growth typically matching or exceeding that of peers. The initial skeletal overgrowth observed in newborns with MPS is thought to result from prenatal accumulation of GAGs. Already during fetal development, excessive storage of GAGs may enhance proliferative signaling through their interaction with growth factors (e.g., FGF, IGF, TGF-β), leading to increased cell and tissue mass and, in consequence, longer birth length [38]. However, this effect is transient. With disease progression, continuous GAG accumulation disrupts cartilage architecture and homeostasis, damages growth plate chondrocytes, and interferes with the regulation of endochondral ossification. As a result, bone maturation and remodeling are impaired, and the initial overgrowth is followed by pronounced growth deficits, leading to the characteristic short stature and skeletal dysplasia observed in infancy and childhood [39]. Despite characteristic skeletal features, which lead to future growth issues, height during infancy remains largely unaffected.

By early childhood, all MPS subtypes invariably fall below normal growth curves. A Polish study demonstrated that boys with Hurler syndrome (MPS I) and severe Hunter syndrome (MPS II) exhibited above-average height at birth but began to diverge around 2½ years [40]. Specifically, Hurler patients dropped below the third percentile by 30 months, while severe MPS II patients did so by 4–5 years. Registry data corroborate that untreated Hurler children show growth deviation as early as 6 months, and attenuated MPS I patients typically diverge around 2 years. By mid-childhood, growth velocity significantly declines across all subtypes.

Pubertal growth spurts in MPSs are markedly reduced or completely absent. Research shows that children with MPS II experience rapid growth during infancy, which sharply declines by age 3, while untreated MPS I patients show virtually no pubertal growth peak. As a result, adult height in individuals with MPS is severely diminished. For instance, males with Morquio A/B average only about 119 cm, untreated Hunter syndrome young adults reach about 125.6 cm, and severely affected Hurler patients average around 110 cm. The severity of the biochemical phenotype correlates with growth outcomes. Severe forms of MPS I and II face earlier and more significant growth failure compared to milder forms. For example, Hurler patients are notably short by age 4, while patients with a milder phenotype remain near normal for a longer period. Morquio A and B patients experience the most extreme short stature, followed by MPS VI and neuropathic MPS I/II, while Sanfilippo (MPS III) patients are comparatively less affected. In conclusion, MPSs demonstrate a clear pattern of above-average birth length, early life growth faltering, and diminished adolescent growth, with both the timing and severity varying distinctly by subtype [41].

Based on our own observations (over 30 years) of growth dynamics in patients with different types of MPS, performed at the Children’s Memorial Health Institute (Warsaw, Poland), we propose standardized mean values for body height z-scores in calendar age classes, see Supplementary Figure S1 and S2.

### 3.3. Skeletal Features

**Figure 1:**
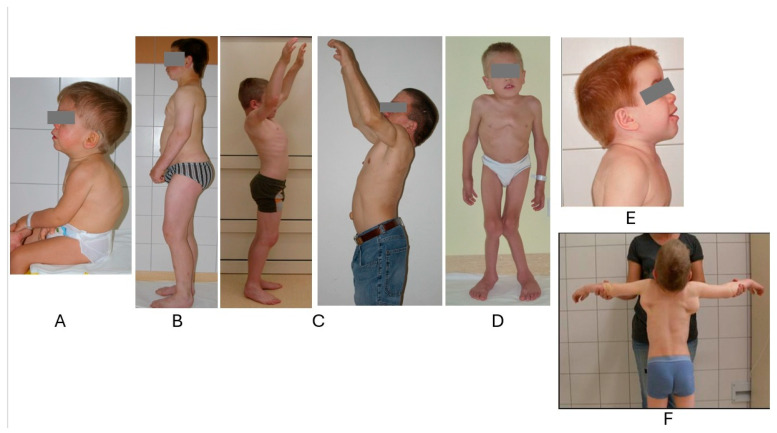
Clinical phenotype of patients with MPS. (**A**) One-year-old patient with MPS IH presenting with a thoracolumbar kyphosis (gibbus). (**B**) Nineteen-year-old patient with MPS IS presenting with normal intelligence. (**C**) Comparison of two patients with MPS II, attenuated form, 12 and 25 years of age. (**D**) Eight-year-old patient with MPS IVA presenting with significant knee and foot valgus, severe sternal protrusion, and kyphosis. (**E**) Coarse facial features with flattened top of the head in MPS I. (**F**) Winged scapula in MPS I.

The skeletal abnormalities observed in patients with MPS are collectively referred to as *dysostosis multiplex.* The most characteristic changes include stiffness and limited mobility of the joints—with the exception of MPS IV—while the most and earliest noticeable are in the shoulder joints as limited abduction (symmetric restriction of shoulder abduction seems very specific to MPS), see Figure 1. Cartilage is the major area of pathology in MPSs, and due to GAG deposition in the chondrocytes, the extracellular matrix of the articular cartilage, the synovia, and the surrounding tissues, MPS patients have stiff joints, contractures, and poor mobility (Figure 1) [35].

Thoracolumbar kyphosis (gibbus) is one of the earliest and most characteristic symptoms of MPS I (Figure 1) [42]. Kyphosis of the lumbar spine is the most common presenting symptom in the severe phenotype and often the only visible abnormality. Joint stiffness without inflammation and kyphosis or gibbus are highly specific presenting signs and symptoms that, on their own, are sufficient to raise suspicion of MPS I.

**Figure 2:**
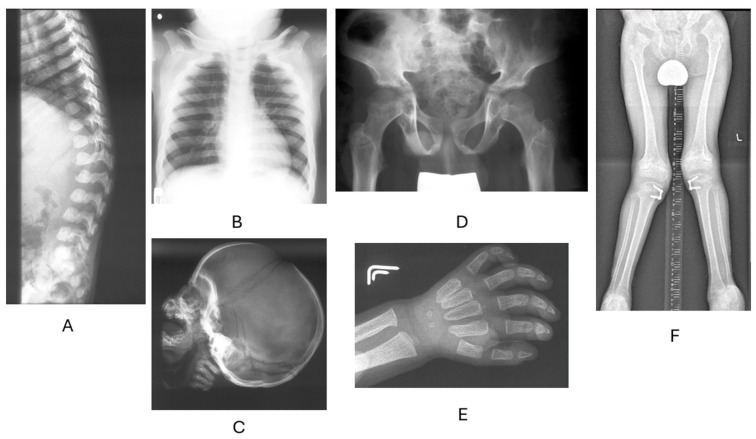
Radiological phenotype of patients with MPS. (**A**) Thoracolumbar kyphosis (gibbus) with a notched anterior part of the vertebral bodies. (**B**) Short, thickened clavicles and paddle-shaped ribs. (**C**) A large skull with a thickened vault and a J-shaped sella turcica. (**D**) Deformed iliac plates, subluxation of the right ilium. (**E**) Sharply pointed metacarpal bones in the proximal part. (**F**) Significant knee valgus in MPS IV. Bilateral medial epiphysiodesis of the proximal tibiae.

There is a high prevalence of carpal tunnel syndrome (CTS) in MPS, and, collectively, MPS disorders are the most common cause of CTS in children [43]. Virtually all patients with MPS I, II, and VI develop CTS. CTS is a consequence of excessive GAG deposition in the perineurium and soft tissues, leading to the compression of the median nerve (at the level of the wrist). It manifests mostly as nighttime pain, numbness, tingling, or a burning sensation, finally leading to the atrophy of the thumb muscles and loss of fine motor functions. CTS symptoms overlap with joint stiffness due to GAG deposition in joints and surrounding tissues. A characteristic hand position in MPS is associated with flexion contractures in the metacarpophalangeal joints, widened proximal phalanges, and widened and short metacarpal bones (Figure 2).

Other skeletal abnormalities include flattened vertebral bodies, oar-shaped ribs, short clavicles, a large skull with a thickened vault, and a J-shaped sella turcica hypoplastic dentary (Figure 2) [44]. An underdeveloped dentary predisposes to atlantoaxial instability, which, together with peri-odontoid soft tissue masses (GAG accumulation behind the odontoid process) and cervical canal stenosis linked to fibrocartilage reactive hypertrophy associated with hypertrophy of the dura and ligamentum flavum, can cause life-threatening spinal cord compression [45]. Clinical findings are often represented by cervical myelopathy symptoms, arising from long-tract compression, including bilateral motor deficits (deterioration of motor function/gait, later tetraparesis/high paraplegia), painful paresthesia, sphincter disturbances (bowel/bladder incontinence), and hyperreflexia [46,47]. Notably, craniovertebral junction instability is a hallmark feature in many different diseases and is mostly recognized in MPS I, IV A and B, and VI, while cervical canal stenosis can often be found in the majority of MPSs, mainly I, II, VI, and VII [48].

Other disorders of the musculoskeletal system manifest themselves as progressive deformities of the spine (scoliosis, excessive kyphosis), deformities of the thorax, early degenerative changes in the joints (especially the hips), and muscle atrophy.

Hip dysplasia in MPSs comes from the variable combination of a flattened acetabulum, hypoplasia of the proximal epiphysis in its medial portion, and coxa valga. Patients with MPS I and II present specific features in hip joint ultrasonography [49]. All patients, regardless of disease progression, present specific ultrasonographic findings such as a significantly thickened synovial joint space with significantly increased echogenicity and no signs of synovitis or increased flow through the joint.

Skeletal symptoms (scoliosis, kyphosis, lumbar lordosis, hip dysplasia) appear later in MPS III and are observed only in a minority of patients.

The characteristic feature of MPS IV is unique skeletal dysplasia, including, among others, a short trunk, kyphoscoliosis, pectus valgus, knock knees, and marked laxity of connective tissue, ligaments, joints, and valves (Figure 1 and Figure 2) [50,51]. Joint hypermobility (of the wrist, in particular) is especially helpful in establishing clinical suspicion, as it is unique to MPS IVA and IVB.

### 3.4. Hernias

Recurrent or persistent hernias are a common early sign of MPSs that should be considered in combination with other signs and symptoms. Hernia repair is the most frequently reported surgical intervention in MPS patients before the age of 5 years [52]. During the first and second year of life, inguinal hernias are much more common among boys suffering from MPS II. Children suffering from MPS I, VI, and VII also experience umbilical hernias [53]. Patients suffering from MPS III and IV more commonly experience hernias compared to the healthy population.

### 3.5. Liver Enlargement

A mildly enlarged liver and spleen can be observed in practically all types of MPS, also in MPS IVA. Only about half of MPS III patients have an enlarged liver volume, and a small percentage have an enlarged spleen volume. Although the enlargement of both of these organs does not affect their function (normal liver transaminases), the enlarged organs can cause pressure on the diaphragm, impacting breathing.

### 3.6. Heart

Cardiac abnormalities occur in all MPS subtypes, with the most common being valvular defects and cardiac hypertrophy [54]. Most studies indicate that this occurs earlier and more frequently in MPS I, II, and VI [55].

The accumulation of DS within the tendinous structures of the heart and valves (especially mitral and aortic) causes their damage (initially discrete, later in life already pronounced valvular insufficiency). The deposition of GAGs in the heart occurs at a very early age and may already start in utero [56,57]. It leads to a substantial thickening, stenosing, or regurgitation of heart valves by a premature accelerated atherosclerotic, immunological–inflammatory, apoptotic, and proliferative change in the cellular valve matrix. The mitral and aortic valves are the most commonly affected, and a progressive cardiac valve pathology is the most prominent and uniform cardiac manifestation (60–90%) of patients with MPS. Valvular stenosis or regurgitation may lead to left atrial and/or left ventricular volume overload, left ventricular dilatation, left ventricular hypertrophy (LVH), and ultimately systolic and diastolic dysfunction []. Conduction abnormalities, including atrial and/or ventricular arrhythmias, have also been reported in patients with MPS; however, most are clinically insignificant.

The severity of cardiologic abnormalities in MPS VI is either minor or severe. A non-classical “cardiac phenotype” has been described in some patients with MPS VI [58,59]. This form manifests later in adulthood with primarily cardiac features such as valvular disease, cardiomyopathy, and/or acute heart failure, although other symptoms (such as musculoskeletal abnormalities) may also be present.

### 3.7. Ear, Nose, and Throat (ENT) and Respiratory Manifestations

ENT and respiratory manifestations are very common and usually appear in the early stage of MPSs, especially types I, II, and VI, see Table 3 [60,61].

Upper airway obstruction has been described for all MPS disorders. Adenotonsillar hypertrophy due to GAG deposition (additionally macroglossia and mandibular abnormalities) leads to progressive upper airway obstruction, obstructive sleep apnea (OSA), and recurrent upper respiratory tract infections (Table 3). MPS patients with clinical symptoms of upper airway obstruction often undergo adenoidectomy and/or tonsillectomy. Previous studies have also reported that children frequently underwent these procedures before the diagnosis of MPS, typically in types I and II [62]. Furthermore, the recurrence rate of adenoid hypertrophy after adenoidectomy in the MPS population is higher than in healthy children [63].

Children with MPS have a high prevalence of OSA—up to 89% of patients with MPS, which could be especially severe in MPS I, despite previous adenotonsillectomy []. Cervical spine instability and odontoid dysplasia may compress the spinal cord and, as a consequence, may induce central sleep apnea.

Mixed hearing loss (conductive and sensorineural deafness) is an almost universal finding in children with MPS [64,65]. The pathomechanism is multifactorial, see Table 3. Otitis media with effusion (OME) in patients with MPS is more common than in the general population as a result of adenoid hypertrophy, GAG accumulation in middle ear fluid, and cranial deformities, which affect Eustachian tubes [66]. The conductive component of hearing loss is usually treated by the insertion of ventilation tubes. However, optimizing the ventilation of the middle ear does not automatically normalize hearing ability; thus, the conductive component may persist after myringotomy. The main treatment for patients with MPS with SHL is hearing aids, like cochlear implants.

Progressive respiratory involvement is caused by disease at multiple levels: macroglossia, diffuse laryngeal mucosal thickening, extrinsic compression of the trachea by GAG deposits, and tracheobronchomalacia. Chest wall deformities including pectus carinatum and kyphoscoliosis can limit lung expansion and produce a restrictive impairment that manifests as a reduction in lung volume. Displacement of the diaphragm into the thoracic cavity may occur due to short stature coupled with hepatic and/or splenic enlargement, further compromising respiratory function. Tracheal involvement in MPS may present as tracheomalacia, resulting from tracheal deformity and weakening of the supporting cartilage, mediastinal compression by GAG deposits, and endoluminal obstruction also by deposits, aggravated by the associated inflammatory reaction [67,68]. These airway abnormalities can result in severe, potentially fatal, difficulties during anesthetic procedures. The trachea can be narrow, tortuous, or occluded by the accumulation of soft tissue [69].

### 3.8. CNS Manifestation

Neurocognitive decline is observed in the neuronopathic forms of MPS I (Hurler), MPS II, and MPS VII, and all MPS III phenotypes, although with varying severity. In neuronopathic MPS phenotypes, the disease is progressive, with a course of relatively normal initial development, followed by a slowing in that development, a halting of further acquisition of skills, the loss of already acquired skills, and finally death [70]. Early detection of cognitive involvement is thus essential in the management and treatment of neurological signs and symptoms in MPS patients [71].

Behavioral abnormalities are most frequently reported in children with MPS, especially MPS III and II, associated with cognitive impairment [72]. Children with MPS IH show relatively mild behavioral problems. Behavioral problems arise early in life (around the age of 2 to 4 years) and include hyperactivity, (hyper)orality/preservative chewing, lack of fear (for danger), disobedience/unresponsiveness to discipline, and destructive behavior.

Sleep disturbances also occur in patients with MPS III and II and include settling difficulties, waking up during the night, insomnia, and early morning awakening, as well as daytime sleeping.

The overall prevalence of epilepsy in MPS is approximately 30%, with the highest in neuronopathic MPS II and MPS III. These patients typically present with generalized tonic–clonic seizures. The incidence of seizures has been found to increase with advancing neurocognitive deterioration.

Focal or diffuse T2-hyperintense signal abnormality in the periventricular white matter is usually the most severe and confluent in MPS III, also being an early finding in MPS I, II, and VII, but tends to occur later and be less extensive in MPS and VI [73]. Brain atrophy is frequently seen in children with MPS and is likely caused by neuronal death, myelin loss, and gliosis. It manifests as enlargement of the ventricular system and subarachnoid spaces. It develops early during the first few years of life in MPS I, II, III, and VII.

Hydrocephalus is a common complication of MPS I and II (less frequent in patients with MPS III, IVA, and VI) and is caused by impaired cerebrospinal fluid resorption secondary to GAG accumulation at the arachnoid granulations [74,75]. It is usually slowly progressive and difficult to differentiate from ventriculomegaly secondary to brain atrophy.

### 3.9. Ocular Features

The most common ocular feature in MPS is corneal clouding, which may impact visual acuity [76,77,78,79]. It is due to the deposition of GAG granules with a yellowish-grey color in all corneal layers. MPS I and MPS VI have a more severe manifestation (it can develop within the first year of life) than MPS IV and MPS VII. The most prominent ocular condition seen in MPS III is retinopathy [77].

### 3.10. Non-Immune Hydrops Fetalis

Non-immune hydrops fetalis (NIHF) was reported in patients with MPS I, IVa, and VII, most often in the course of MPS VII. In the most severe cases, MPS VII presents as NIHF and may result in stillbirth or death within the first few days/weeks of life [80,81]. The presence of neonatal hydrops fetalis does not, by itself, predict the eventual severity of the disease. However, affected patients show a wide range of clinical variability, from early, severe, multisystem manifestations to a milder phenotype with later onset and normal or near-normal intelligence.

## 4. Biochemical Diagnostics

The laboratory diagnosis of MPSs relies on the identification of stored material (HS, DS, KS, CS, and hyaluronan—see Table 1), demonstrating the absence of enzyme activity, and detection of pathogenic variants in relevant genes.

Traditionally, the laboratory workflow contains the following steps: (i) non-specific screening test for GAG excretion in urine (quantitative, semi-quantitative); (ii) differentiation of GAGs in urine using electrophoresis or thin-layer chromatography (the type of disease can be suggested at this step); (iii) demonstration of enzyme inactivity; and (iv) identification of pathogenic variants in specific genes.

The most popular (and also easiest) qualitative screening test in urine is the Berry spot test with toluidine blue. The results of this test only allow the identification of individuals with increased GAG excretion, but the exact amount or type of excreted compounds cannot be determined.

The quantity of GAGs in urine can be measured by means of (i) a test with cetylpyridinium chloride (CPC) or (ii) a test with dimethyl-methylene blue (DMB).

The results of quantitative analyses reveal the scale of excretion. These tests can also be used for the monitoring of treatment progress. The excretion of urinary GAGs is related to the age of a patient, changes in the excretion levels during the day, and dietary compounds and medicines [82].

False negative results are possible in patients with MPS III (Sanfilippo disease) or MPS IV (Morquio disease) [82].

False positive results can be obtained in patients with excessive connective tissue destruction, rickets, malabsorption syndrome with gross osteomalacia, malignant disorders (including leukemia), disseminated lupus erythematosus, rheumatoid arthritis, and Marfan syndrome or in neonates [82]. Heparin and chemical compounds used in paper diapers can also give false positive or difficult-to-interpret results [82]. False positive results of quantitative tests can be verified by qualitative tests.

The type of excreted GAG can be determined by the following qualitative analyses: (i) thin-layer chromatography (TLC); (ii) electrophoresis (one- or two-dimensional); and (iii) high-performance liquid chromatography (HPLC).

In qualitative tests, HS may be slightly elevated in cases of multiple sulfatase deficiency. MPS VII is difficult to identify (various patterns of GAG excretion are observed; it may resemble the pattern of a healthy control). KS excretion in patients with MPS IV may be low, making its detection difficult [82].

In high-throughput laboratories, either qualitative or quantitative analyses are performed by means of tandem mass spectrometry (MS/MS) in the serum/plasma or dried blood spots (DBSs).

On the basis of the results of qualitative tests, enzymatic analyses can be projected.

Enzymatic tests use various biological sources of enzymes (so-called test material), including (i) isolated blood leukocytes; (ii) plasma or serum; (iii) cultured skin fibroblasts; (iv) cultured amniocytes or chorionic villi (for prenatal testing); and (iv) DBSs.

Enzymatic activity tests are performed with the use of substrates, which can be (i) natural isotope-labeled compounds—these are not commercially available, and they require technological knowledge and technical facilities for independent production— and (ii) artificial chromogenic (e.g., p-nitrophenol derivatives) or fluorogenic (e.g., 4-methylumbelliferone derivatives) compounds, which are commercially available.

In the case of artificial substrates, it is important to take into consideration the phenomenon of pseudo-deficiency in enzymatic activity, which occurs in carriers of polymorphic variants in genes encoding lysosomal enzyme proteins. As a result, newly synthesized proteins may have an altered spatial structure or may be unstable, but they retain sufficient activity to ensure normal metabolic turnover in vivo. However, in vitro, the altered protein molecules do not catalyze the reaction with the artificial substrate, which is detected as a lack (deficiency) of activity, which may suggest a diagnosis of the disease. In reality, however, as already mentioned, the enzyme is active in vivo, so the deficiency is apparent (i.e., it is a pseudo-deficiency, a false deficiency). Pseudo-deficiencies in enzyme activities can lead to misdiagnosis, which should always be kept in mind during the diagnostic process and confirmed by additional biochemical tests (e.g., demonstrating substrate storage) or molecular tests (detection of polymorphic variants).

Complete deficiency or a very low residual enzyme activity is considered the definitive diagnosis, which is usually confirmed by DNA analysis. Knowledge of the molecular basis of the disease in a given family is also useful for genetic counseling. In cases of high residual activity, the diagnosis is confirmed by mutation analysis and demonstration of substrate storage.

In high-throughput laboratories, levels of GAG-derived disaccharides and enzyme activities in blood or DBSs are measured with HPLC-MS/MS. Other methods used in such laboratories include (i) digital microfluidic cartridges using a single DBS punch to perform a multiplex fluorometric enzymatic assay; (ii) immune quantification of lysosomal enzyme protein levels, as opposed to enzyme activity levels; and (iii) the lab-on-a chip technique.

Alternative methods for diagnostics of MPS include (i) capillary electrophoresis (CE), where digested GAGs are detected by ultraviolet spectroscopy or mass spectrometry (MS); (ii) microchip electrophoresis; (iii) ELISA tests for DS and HS estimation; and (iv) hair morphology using an electron microscope [83].

## 5. Molecular Diagnostics

MPS-causing genes exhibit extensive allelic heterogeneity, with pathogenic changes ranging from single-nucleotide variants (SNVs) and small insertions or deletions (indels) that may induce frameshifts or premature stop codons to larger structural alterations such as copy-number variations (CNVs). Severe clinical phenotypes are most often associated with loss-of-function (LOF) variants that result in premature termination codons and subsequent degradation of the transcript via nonsense-mediated decay (NMD). In contrast, missense variants typically lead to a partial reduction in enzyme activity by altering the protein’s three-dimensional conformation, affecting folding, stability, or interaction with essential cofactors or substrates.

Table 4 presents a summary of the most frequently reported variants in MPSs, including their impact on the phenotype.

The diagnostic strategy may involve targeted direct variant testing, whole-gene sequencing (using Sanger sequencing), or differential testing with a multigene panel or phenotype-focused exome analysis (using NGS methods) [84]. If only a single variant is identified, further molecular testing should be performed, extending the analysis to include CNV detection (via NGS or MLPA), as well as the evaluation of deep intronic regions that may affect splicing.

The classification of genetic variants should be performed according to the American College of Medical Genetics and Genomics and the Association for Molecular Pathology (ACMG/AMP) guidelines [85]. This evaluation should integrate data from publicly available resources such as ClinVar, alongside evidence from the peer-reviewed scientific literature. Tomanin et al. reviewed all variants in the *ARSB* gene in patients with MPS VI reported in the literature and in public databases . Only 18% of them had been previously reported in genetic databases in association with supporting evidence or clinical classification [86].

Molecular characterization of MPS patients has revealed a high incidence of particular variants of different populations or geographical origins, facilitating accurate molecular diagnosis of the disease.

### 5.1. MPS I

Severe MPS I most frequently results from pathogenic IDUA variants that lead to a complete loss of enzyme activity, including c.1205G>A (p.Trp402Ter) and c.208C>T (p.Gln70Ter). In contrast, attenuated phenotypes are usually observed in individuals carrying at least one milder allele, often a missense variant, which allows partial residual enzyme activity. In European populations, c.1205G>A (p.Trp402Ter) and c.208C>T (p.Gln70Ter) are the most prevalent pathogenic alleles, with at least one of these variants identified in 35–60% of patients of Caucasian origin. Population-specific alleles have also been reported, including c.266G>A (p.Arg89Gln) and c.613_617dupTGCTC in Japan and p.Pro533Arg in Morocco, the latter accounting for ~90% of affected individuals in that country [87,88,89].

### 5.2. MPS II

While MPS II primarily affects males due to its X-linked recessive inheritance and the presence of a single X chromosome, affected females have also been reported [10]. In females, disease manifestation may occur through mechanisms such as skewed X-chromosome inactivation, leading to preferential silencing of the normal *IDS* allele and consequent reduction in iduronate-2-sulfatase activity. Additionally, functional hemizygosity in heterozygous females, where one pathogenic *IDS* allele is present and the other is rendered inactive, can result in milder clinical features. Rarely, biallelic inheritance of pathogenic *IDS* variants may occur in females, contributing to more pronounced disease phenotypes. Most affected females were found to carry the pathogenic allele on the maternally inherited X chromosome [90].

Zanett et al. (2024) performed a comprehensive analysis of *IDS* gene variants reported across the scientific literature and genetic variant databases [91]. Among the most commonly reported pathogenic variants in the global population were the following: c.1122C>T (p.Glu375_Gly394del), c.1403G>A (p.Arg468Gln), c.1402C>T (p.Arg468Trp), c.998C>T (p.Ser333Leu), c.1327C>T (p.Arg443Ter), c.262C>T (p.Arg88Cys), c.263G>A (p.Arg88His), c.253G>A (p.Ala85Thr), c.257C>T (p.Pro86Leu), and c.514C>T (p.Arg172Ter). These variants were identified in about 20% of MPS II patients.

Further genetic causes of the disease are partial deletions, large deletion–insertions, or complete deletions of the *IDS* gene. CNVs are estimated to be responsible for the disease in approximately 20% of all cases of MPS II. Patients with full deletions and complex rearrangements of the *IDS* gene generally present with a severe clinical phenotype [91]. The presence of a pseudogene called *IDSP1,* located 3.9 kb from *IDS,* complicates certain genetic testing methods. A recombination event between the *IDS* gene and the *IDSP1* pseudogene is the cause of disease in an estimated 13% of MPS II patients. In most of them, this recombination results in an inversion [91,92]. The presence of this rearrangement is typically associated with a severe phenotype.

### 5.3. MPS III

MPS IIIA is caused by pathogenic or likely pathogenic variants in the *SGSH* gene, which comprises eight exons spanning approximately 11 kb. Over 200 pathogenic variants have been identified in the *SGSH* gene [93]. The severe, rapidly progressive phenotype is typically observed in homozygotes or compound heterozygotes for truncating or certain missense variants, such as c.1080del (p.Val361SerfsTer52), c.734G>A (p.Arg245His), c.220C>T (p.Arg74Cys), c.1139A>G (p.Gln380Arg), and c.197C>G (p.Ser66Trp) [94,95]. In contrast, c.892T>C (p.Ser298Pro) is associated with a slower-progressing or attenuated phenotype, indicating residual enzyme activity. The pathogenicity of these variants has also been confirmed through functional studies [95,96]. Several pathogenic variants demonstrate population-specific prevalence: c.220C>T (p.Arg74Cys) is more common in Poland (detected in 56% of MPS III cases), c.734G>A (p.Arg245His) is frequently reported in the Netherlands, Germany, and Australia, c.197C>G (p.Ser66Trp) is prevalent in Italy, and c.1080del (p.Val361SerfsTer52) is observed predominantly in Spain [97].

MPS IIIB results from pathogenic variants in the *NAGLU* gene, which contains six exons and spans 8.3 kb. Almost all nonsense and frameshift variants are considered pathogenic. Most reported variants occur at low frequencies or are unique to individual families [98].

MPS IIIC is caused by dysfunction of heparan-α-glucosaminide N-acetyltransferase, an enzyme encoded by the *HGSNAT* gene, which comprises 18 exons [99]. MPS IIID is caused by defects in the *GNS* gene, which encodes the enzyme N-acetylglucosamine-6-sulfatase and comprises 14 exons. For both MPS IIIC and MPS IIID, pathogenic variants are distributed throughout the *HGSNAT* and *GNS* coding sequences, respectively, with no clearly defined mutational hotspots. Loss-of-function variants are generally associated with pathogenicity and more severe phenotypes. Due to the rarity of these subtypes, no population- or region-specific recurrent variants have been described to date [100,101].

### 5.4. MPS IV

MPS IV is a genetically heterogeneous disorder caused by biallelic pathogenic variants in either the *GALNS* gene (MPS IVA) or the *GLB1* gene (MPS IVB).

The *GALNS* gene encodes N-acetylgalactosamine-6-sulfatase, a lysosomal hydrolase involved in the catabolism of keratan sulfate and chondroitin-6-sulfate. To date, nearly 900 pathogenic/likely pathogenic *GALNS* variants have been reported. These include missense, nonsense, canonical splicing, and deep intronic variants, as well as small and gross insertions, duplications, deletions, or complex genomics rearrangements [102]. Among the most frequently observed pathogenic variants is c.1156C>T (p.Arg386Cys), detected in approximately 9% of MPS IVA patients [103]. Variants typically associated with a severe phenotype include c.1520G>T (p.Cys507Phe) and c.29G>A (p.Trp10Ter), whereas milder clinical manifestations have been reported for c.178G>A (p.Asp60Asn), c.612C>G (p.Asn204Lys), and c.776G>A (p.Arg259Gln) [104]. In approximately 16% of patients, the second expected disease-causing *GALNS* variant cannot be reliably identified using standard molecular diagnostic approaches targeting the coding sequence and exon–intron boundaries [105]. In such cases, further investigation should encompass the analysis of deep intronic regions and transcript-based studies (e.g., RNA sequencing) to detect potential splicing defects or pathogenic alterations in non-coding regions [97,106].

Pathogenic variants in the *GLB1* gene lead to two distinct clinical disorders: GM1 gangliosidosis and MPS IVB (Morquio B disease). Despite the shared genetic basis, the pathophysiology and clinical features of MPS IVB and GM1 gangliosidosis differ significantly. Although both GM1 gangliosidosis and MPS IVB disease arise from pathogenic variants in the *GLB1* gene, distinct molecular characteristics and mutation biases typically underlie each condition. In GM1 gangliosidosis, pathogenic variants tend to result in severe or complete loss of β-galactosidase activity, often affecting the catalytic or elastin-binding domains, leading to accumulation of GM1 gangliosides in neuronal tissue and early-onset neurodegeneration [106]. In contrast, MPS IVB is usually caused by missense variants that preserve partial β-galactosidase activity, sufficient to degrade small amounts of gangliosides but insufficient for effective catabolism of KS in the cartilage [106]. Some variants (such as c.602G>A (p.Arg201His) and c.203G>A (p.Arg68Gln)) have been reported in both disease phenotypes, suggesting that differential substrate specificity or residual enzyme activity and a modified genetic background can modulate clinical expression. These genotype–phenotype distinctions highlight that the variant type and functional impact in the *GLB1* gene determine whether the disease manifests predominantly as neurological (GM1) or skeletal (MPS IVB).

### 5.5. MPS VI

The ARSB gene, which encodes arylsulfatase β, contains eight exons and spans about 206 kb. Disease-causing variants in this gene are distributed throughout its length rather than clustered in specific regions. Genotype–phenotype correlations among homozygotes were generally inconclusive for most variants; however, certain types, such as large deletions, nonsense and frameshift mutations, and select missense changes, tended to be linked with a more rapidly progressive disease course [87,107,108].

### 5.6. MPS VII

The *GUSB* gene encodes β-glucuronidase, spans approximately 21 kb of genomic DNA, and consists of 12 exons. Pathogenic *GUSB* variants are distributed throughout the entire gene. Most of the variants are SNVs or small indels. There are no regions that can be defined as hotspots. Nonsense variants and small deletions are mostly associated with severe phenotypes [54]. Founder variants include c.1244C>T (p.Pro415Leu) and two *in cis* variants c.[1244C>T;1222C>T] (p.[Pro415Leu;Pro408Ser]) in the Mexican population, c.1856C>T (p.Ala619Val) in the Japanese population, and c.526C>T (p.Leu176Phe) in the Brazilian population, with almost all affected individuals being homozygous [109,110].

### 5.7. MPS IX

Mucopolysaccharidosis type IX (MPS IX) is caused by homozygous or compound heterozygous variants in the gene *HYAL1*, which encodes the enzyme hyaluronidase-1. The gene spans approximately 32 kb and consists of six exons. To date, 40 variants have been reported as pathogenic and likely pathogenic. Most of them are nonsense or frameshift variants (98%). Due to the fact that the disease is relatively rare, no correlation has been observed between the severity of clinical symptoms and the type of variant or the frequency of the variant in a given population.

### 5.8. MPS X

Mucopolysaccharidosis type X (MPS X) is caused by homozygous or compound heterozygous pathogenic variants in the *ARSK* gene, encoding arylsulfatase K. In two sib pairs reported by Verheyen et al. (2022), homozygous *ARSK* variants were identified, p.ArgR84Cys (resulting in reduced enzymatic activity) and p.Leu187Ter, resulting in the absence of protein expression[111]. Rustad et al. identified a homozygous nonsense *ARSK* variant (p.Tyr417Ter) in two other sibs with MPS10. The variant was located in the penultimate exon and was predicted to result in nonsense-mediated decay. To date, ten ARSK-related MPS X patients from six families have been reported, with a median age at presentation of 9.5 years [112].

### 5.9. MPS-Plus

To date, nearly all reported MPS-PS patients have come from the Yakut population in Russia, plus two cases from Turkey; it is to be noted that Yakuts and Turks belong to the Turkic ethnic group. All of these individuals were homozygous for the c.1492C>T (p.Arg251Glu) variant in the *VPS33A* gene. Only two additional patients have been described with a different *VPS33A* variant, c.599G>C (p.Arg200Pro), which was long thought to be unique to MPS-PS [8]. Notably, those carrying p.Arg200Pro exhibit a milder clinical course and survive significantly longer than patients homozygous for p.Arg251Glu.

## 6. Brief Overview of Available and Emerging Therapies for MPSs

Two main treatment regimens for MPS patients are available in practice: hematopoietic stem cell transplantation (HSCT) and enzyme replacement therapy (ERT) [112,113]. The first bone marrow transplantation in MPS was performed in a 1-year-old boy with MPS IH in 1981. HSCT is now the gold-standard treatment for MPS IH and an acceptable and favorable treatment option for MPS II; however, the method of treatment depends on the patient’s age at diagnosis and the stage (severity) of disease. HSCT has also been shown to be effective in some patients with MPS types IVA, VI, and VII [114]. The extent to which the treatment is effective depends on the age of the patient and the disease stage at the time of the procedure, the type of MPS, the type of donor, and the preparative regimen [114]. HSCT has not been able to significantly correct clinical manifestations of the disease in the bone or cornea, cardiac valvular abnormalities, or any preexisting cognitive and intellectual effects of the disease [114].

The introduction of ERT, which involves the intravenous administration of the active form of the enzyme (usually its recombinant form), which is otherwise defective in the patient’s cells, has been a breakthrough in treatment of MPS. MPS I was the first to be treated with ERT (Aldurazyme), which has been available since 2003. Over the past dozen or so years, ERT has been developed also for MPS types II, IVA, VI, and VII [115]. There is general agreement that ERT is effective in reducing urinary GAGs and liver and spleen volume, while its effectiveness regarding the trachea and bronchi, joints, hearing, and eyes is definitely poor, probably due to limited penetration in specific tissues [115]. ERT also does not cross the blood–brain barrier (BBB), and this fact especially concerns patients with severe (neuronopathic) forms of MPS. To overcome this limitation, several proposals were developed, including the use of fusion proteins (composed of the enzyme and a carrier protein) capable of crossing the BBB (thus working as “Trojan horses” to deliver the therapeutic enzyme to the brain) [116], intrathecal delivery of the enzyme [117], and the use of engineered autologous B cells, though studies are still at the experimental or clinical trial [118] stages. All patients treated with recombinant enzymes develop anti-drug antibodies, but their role in ERT tolerance and effectiveness has not been well defined yet [115].

Having HSCT and ERT as approved therapeutical procedures, it was a natural goal to combine these two methods of treatment of MPS patients. The first attempt at such a combination was reported as early as in 2005, when 12 patients with MPS I were investigated. Eleven of them completed the study, which gave promising results, but the results were not conclusive enough to indicate unequivocally advances over any single therapy [119]. A subsequent study indicated that using ERT prior to HSCT is unlikely to significantly change the engraftment efficiency [120]. However, when a combination therapy consisting of ERT and HSCT was started in newborn MPS I mice, added benefits (relative to single therapeutic approaches) were observed, including reduced anomalies in the skeleton and decreased neuroinflammation [121]. Quite similar benefits were then observed in MPS I patients [122]. Although most studies on combining ERT with HSCT were conducted in MPS I, similar efficacy was also observed in MPS VI (excluding neurological symptoms which are absent in this MPS type) [123].

Due to the need to develop a therapy for the neuronopathic forms, an alternative approach has been proposed—substrate reduction therapy (SRT). In theory, this method involves inhibiting GAG biosynthesis when degradation of these compounds is impaired, which allows the balance between GAG formation and removal to be restored. One of the substances used in SRT, aiming to cross the BBB, was trihydroxyisoflavone (genistein), a natural compound from the isoflavone group [124]. However, despite the high efficacy of this method, demonstrated in cellular and animal models of MPS, clinical trials showed a reduction in GAG levels but limited clinical efficacy [125,126,127,128,129,130,131].

Gene therapy seems to be a hope for patients suffering from genetic diseases, including MPS. The idea of this kind of therapy is to provide a functional gene to cells of patients suffering from diseases caused by the presence of pathogenic variant(s) of this gene. In most gene therapy approaches, the viral vectors used as viruses are naturally occurring carriers of genetic material capable of effectively delivering DNA or RNA into animal and human cells. The stage of development of gene therapy for MPS has been summarized recently [132]. Among the most frequently used specific approaches there are ex vivo methods (based on transplantation of hematopoietic stem progenitor cells which are modified to carry an active allele of the desired gene) and in vivo methods (using direct infections of patients with viral vectors bearing the therapeutic gene). Various types of adeno-associated viruses (AAVs) and lentiviral vectors are the most frequently used vehicles to carry therapeutic genes for MPS. There are different ways of applying such vectors, including intravenous, intracerebral, intracisternal, and intracerebroventricular administration. Currently, the most advanced studies on development of gene therapy for MPSs are works focused on types I, II, IIIA, and VI [132]. Some studies are at the stage of interventional clinical trials, while in the case of MPS I, II, and IIIA, there are phase III clinical trials ongoing [133].

The most important challenges in developing effective gene therapies are (i) problems with effective delivery of vectors carrying therapeutic genes into a sufficient number of the patient’s cells, (ii) difficulties in obtaining stable expression of the therapeutic gene for a long time, and (iii) issues with ensuring high levels of safety of the therapeutic procedure. The last point has been highlighted recently, as cases of severe adverse effects, like myelodysplastic syndrome or even death, were reported in recent years [133]. The challenges indicated above are being addressed by the use of various modern molecular methods. They can be exemplified by (but are not limited to) the use of non-viral vectors (like plasmids or transposons), which are characterized by elevated safety relative to the viral ones [134], employing the CRISPR/Cas9 technology to introduce genetic modifications [135], and approaches to modify genomes with other tools [136]. Especially intriguing approaches can be identified as the use of CRISPR/nCas9-based gene therapy to edit genomes of CD34^+^ cells in order to achieve cross-correction between cells (such an approach was tested in an MPS IVA model) [137] or employment of AAV-mediated homology-independent targeted integration of desired genes by CRISPR/Cas9 [138]. All of the abovementioned and other attempts, summarized recently [139], give hope for developing fully effective therapies for MPSs which are, unfortunately, still absent.

In summary, despite considerable progress in developing therapeutical approaches for MPSs (summarized in a general way, providing an overview of the current stage, in Table 5), no currently available therapy is fully effective, and apart from HSCT and ERT, different types of drugs are still at the preclinical stages or—at best—undergoing clinical trials. On the other hand, no treatment methods have been registered so far for MPS types IIIA, IIIB, IIIC, IIID, IVB, IX, X, and MPS-PS [1,14]. Although the underlying cause of MPSs has been known for many years, the precise molecular mechanisms of this group of diseases still remain somewhat unclear.

## 7. Conclusions

**Figure 3:**
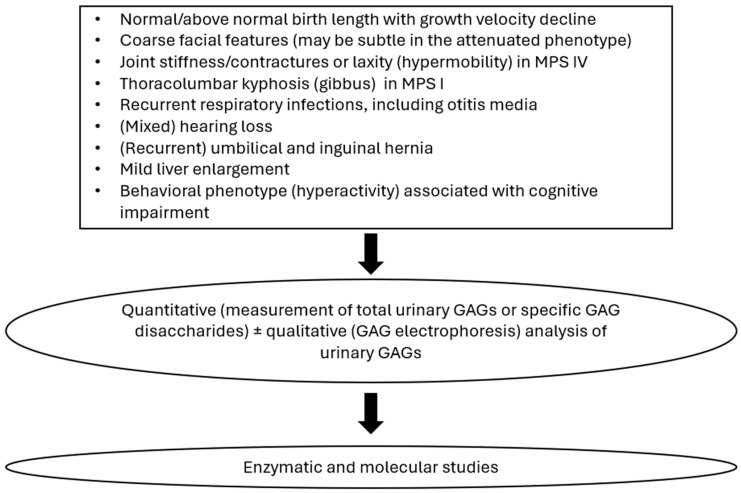
Schematic overview of the early diagnosis of MPSs.

In every patient with clinical signs and symptoms suggesting MPS, as depicted in Figure 3, we recommend the subsequent biochemical and molecular diagnostics. The first step of analytical action should involve the analysis of urinary GAGs, which can be quantitative (measurement of total urinary GAGs or specific GAG disaccharides) or qualitative (GAG electrophoresis to analyze specific GAGs excreted). Neither quantitative nor qualitative methods can diagnose a specific lysosomal enzyme deficiency; however, an abnormality detected by either or both methods indicates the likely presence of MPS. The results of GAG electrophoresis (or other quantitative method) should be the basis for the second step of analytical action, i.e., the measurement of enzymatic activity and molecular testing. Proper early diagnosis is crucial, especially in MPS types for which therapeutic options are already available. However, such diagnosis is also important in other MPS types to provide the best possible care and to avoid ineffective treatments which cause adverse effects, which are possible in the case of misdiagnosis.

One of the major difficulties is the early diagnosis of MPSs. Newborn screening (NBS) methods have become increasingly available for MPSs (available in some regions of North and South America, Asia, and Europe). With the increasing availability of new treatment regimens with a better clinical outcome when started early in life and the availability of combined multiplex assays for MPSs, more new pilot newborn screening programs will appear in the near future.

## Figures and Tables

**Table 1:** Characteristics of MPS- and related-disorder-causing genes.

Gene	HGNC ID	Cytogenetic Localization	Reference Sequence *	Disease	Accumulated GAG	MIM Number	MOI
*IDUA*	5391	4p16.3	NM_000203.5	MPS IH (Hurler)	HS + DS	60014	AR
				MPS IS (Scheie)		607015	AR
				MPS IH/S (Hurler–Scheie)		607016	AR
*IDS*	5389	Xq28	NM_000202.8	MPS II (Hunter syndrome)	HS + DS	309900	XLR
*SGSH*	10818	17q25.3	NM_000199.5	MPS IIIA (Sanfilippo A)	HS	252900	AR
*NAGLU*	7632	17q21.2	NM_000263.4	MPS IIIB (Sanfilippo B)		252920	AR
*HGSNAT*	26527	8p11.21-p11.1	NM_152419.3	MPS IIIC (Sanfilippo C)		252930	AR
*GNS*	4422	12q14.3	NM_002076.4	MPS IIID		252940	AR
*ARSG*	610008	17q24.2	NM_014960.3	MPS IIIE—subtype found only in animal models	HS	618144 (Usher disease type IV)	AR
*GALNS*	4122	16q24.3	NM_000512.5	MPS IVA	C6S + KS	253000	AR
*GLB1*	4298	3p22.3	NM_000404.4	MPS IVB	KS	253010	AR
*ARSB*	714	5q14.1	NM_000046.5	MPS VI (Maroteaux–Lamy Syndrome)	DS + C4S	253200	AR
*GUSB*	4696	7q11.21	NM_000181.4	MPS VII (Sly Syndrome)	HS + DS + C4S + C6S	253220	AR
*HYAL1*	5320	3p21.31	NM_033159.4	MPS IX (Natowicz Syndrome)	Hyaluronan	601492	AR
*ARSK*	25239	5q15	NM_198150.3	MPS X	DS 2-O-sulfo-glucuronate	619698	AR
*VPS33A*	18179	12q24.31	NM_022916.4	MPS-plus ** syndrome	DS ± HS ± KS or normal (profile is highly variable)	617303	AR

Abbreviations: DS—dermatan sulfate; HS—heparan sulfate; KS—keratan sulfate; C6S—chondroitin-6-sulfate; C4S—chondroitin-4-sulfate; MOI—mode of inheritance; AR—autosomal recessive; XLR—X-linked recessive; * refers to MANE Select sequence (accessed March 2025). ** MPS-PS resembles MPS due to GAG accumulation and some symptoms, while enzymes involved in GAG degradation remain unchanged.

**Table 2:** Mucopolysaccharidoses and related disorders—summary of biochemical and genetic phenotypes.

Clinical Features	MPS Type								
	MPS I	MPS II	MPS III	MPS IV	MPS VI	MPS VII	MPS IX	MPS X	MPS-PS
Coarse facial features	+	+	+/−	+	+	+	+/−	+/−	+/−
Hypertrichosis	+	+	+		+	+			
Hearing impairment	++	++	+	+	++	++		+	+/−
Macrocephaly	+	+	+		+	+		+/−	
Corneal clouding	++	+/−		+	++	+		+/−	
Short stature	+	+		++	++	+	+/−	+	+/−
Joint stiffness	++	++	+/−		++	++		+/−	+/−
Thoracolumbar kyphosis	++	+	Lordosis	+	+	+			
Hip dysplasia	++	+	+	++	+	+	+/−	+/−	
Carpal tunnel syndrome	++	++	+/−		++	+			
Joint laxity				++				+/−	
Cardiac valve thickening	++	++	+	+	++	+			+/−
Cognitive impairment	+	+	++			+			
Hydrocephalus/ventriculomegaly	++	++	+	+/−	+	+			
Spinal stenosis	+	+	+/−	++	+				
Recurrent respiratory tract infections	++	++	+	+	++	++	+	+/−	+/−
Upper airway obstruction	++	++	+/−	+	++	++			+/−
Lower airway obstruction	++	++	+/−	+	++	++			+/−
Restrictive lung disease	++	+	+/−	++	+	+			
Liver enlargement	+	+	+/−		+	+			+/−
Inguinal hernia	++	++	+		++	++			
Fetal ascites	+			+		++			+/−

Abbreviations: “++” often present; “+” present; “+/−” can be present.

**Table 3:** Respiratory manifestation of MPSs.

Organ	Signs and Symptoms
Mouth	Thickened lips
	Gingival hypertrophy
	Macroglossia
	Tonsillar hypertrophy
	Restriction of the mouth opening (decreased temporo-mandibular joint mobility)
	Sore throat—swelling of the mucosa
	Lots of mucus
Nose	Depressed nasal bridge, wide nasal alae
	Restricted nasal airflow
	Tonsillar hypertrophy
	Recurrent nose infections, chronic rhinosinusitis
Larynx	Stridor, laryngomalacia (deposits in epiglottis and decreased muscle tone)
	Deformities of the epiglottis and cricoid cartilages
	Narrowing of the larynx
Outer and middle ear	Narrowing of the external auditory canals
	Chronic otitis externa
	Middle ear effusion, chronic inflammation of the middle ear
	Deformation of the ossicles, especially the stapes
	Thickened mucosa in the middle ear
Inner ear	Degeneration of the organ of Corti
	Lack of neurons, GAGs in the spiral ganglion
	Distended, congested vessels in the stria vascularis
Trachea	Tracheal distortion
	Tracheal narrowing
	Tracheomalacia
	Airway collapse
	Complications of endotracheal intubation

**Table 4:** The most common pathological variants of genes resulting in different MPS types.

MPS Type	Gene	Variants	
		Associated with Severe Phenotype	Associated with Attenuated/Mild Phenotype
I	*IDUA*	c.208C>T (p.Gln70Ter) c.1205G>A (p.Trp402Ter)	c.1469T>C (p.Leu490Pro) c.266G>A (p.Arg89Gln) c.1598C>G (p.Pro533Arg) c.700C>T (p.Arg234Cys) c.613_617dupTGCTC (p.Glu207AlafsTer29) ^V^
II	*IDS*	c.1403G>A (p.Arg468Gln) c.1402C>T (p.Arg468Trp) c.998C>T (p.Ser333Leu) c.257C>T (p.(Pro86Leu) c.514C>T (p.(Arg172Ter) c.1122C>T (p.Glu375_Gly394del) ^V^ Recombination with IDS pseudogene (*IDSP1*) causing inversions/complex rearrangements	c.1327C>T (p.Arg443Ter) c.253G>A (p.(Ala85Thr)) c.262C>T (p.Arg88Cys) ^V^ c.263G>A (p.(Arg88His) ^V^
IIIA	*SGSH*	c.220C>T (p.Arg74Cys) c.1139A>G (p.Gln380Arg) c.197C>G (p.Ser66Trp) c.1080del (p.Val361fs)	c.892T>C (p.Ser298Pro) c.734G>A (p.Arg245His) ^V^
IIIB	*NAGLU*	c.1834A>G (p.Ser612Gly) c.889C>T (p.Arg297Ter) c.419A>G (p.Tyr140Cys) c.1562C>T (p.Pro521Leu) c.358G>T (p.Glu120Ter)	c.1843C>T (p.Arg615Cys) c.1694G>A (p.Arg565Gln) c.700C>T (p.Arg234Cys)
IIIC	*HGSNAT*	LOF variants distributed across exons are usually severe	Some missense variants with partial activity reported in attenuated cases (no single dominant founder)
IIID	*GNS*	Truncating and canonical splice variants usually pathogenic → severe disease	Several missense variants reported with milder course; overall fewer cases, so correlations are limited
IVA	*GALNS*	c.1156C>T (p.Arg386Cys) c.29G>A (p.Trp10Ter) c.1520G>T (p.Cys507Phe)	c.178G>A (p.Asp60Asn) c.612C>G (p.Asn204Lys) c.776G>A (p.Arg259Gln)
IVB	*GLB1*	Variants that disrupt catalytic site or cause truncation → GM1/neurologic severe disease; some variants give MPS IVB skeletal-predominant phenotype (e.g., specific missense in KS-processing region); compound heterozygous states combining alleles with differing effects can produce blended phenotypes with both neurological and skeletal features	
VI	*ARSB*	Large deletions, nonsense, frameshifts, and some missense abolishing enzymes → more rapidly progressive disease	Missense variants with residual ASB activity associated with attenuated/osteoarticular phenotypes
VII	*GUSB*	Nonsense/truncating variants and some missense → severe (hydrops fetalis/perinatal fatal)	c.1244C>T, p.(Pro415Leu) c.1856C>T, p.(Ala619Val)
IX	*HYAL1*	Very rare—reported nonsense/deleterious variants in reported cases producing mild phenotype	Very rare—reported nonsense/deleterious variants in reported cases producing mild phenotype: p.Glu268Lys; c. 1361del37ins14, p
X	*ARSK*	Ten cases published so far; mostly attenuated phenotypes	Ten cases published so far; mostly attenuated cases including homozygosity for p.ArgR84Cys, p.Leu187Ter, and p.Tyr417Ter
MPS-plus	*VPS33A*	c.1492C>T, p.(Arg498Trp)	c.599G>C, p.(Arg200Pro)

Abbreviations: ^V^—variable phenotype, dependent on the second variant.

**Table 5:** The major therapies for mucopolysaccharidoses—a general overview.

Therapy (Abbreviation)	Current Stage in MPS	General Principle(s)	Major Limitation(s)
Hematopoietic stem cell transplantation (HSCT)	Approved in some MPS types (I, II, IVA, VI, VII)	Functional enzyme produced by transplanted cells can cross-correct the enzyme deficit in MPS patient cells	Poor penetration to some organs and tissues (e.g., brain, heart, bone)
			Effective only when provided before the 2nd year of age
			Inefficient in some MPS types (e.g., III)
Enzyme replacement therapy (ERT)	Approved in some MPS types (I, II, IVA, VII, VII)	Recombinant (active) enzyme, administered intravenously, can cross-correct the enzyme deficit in MPS patient cells	Poor penetration to some organs and tissues (e.g., brain, bone), causing inefficiency in improving symptoms related to the brain, bones, joints, and trachea
Substrate reduction therapy (SRT)	Experimental/clinical trials	Slowing down synthesis of GAGs by using small molecule(s), thus restoring the balance between GAG synthesis and degradation rates	Limited clinical efficacy, despite decreasing GAG levels in urine, plasma, and cerebrospinal fluid
Classical gene therapy (GT)	Experimental/clinical trials	Delivery of the functional gene into cells of MPS patients using viral or non-viral (e.g., plasmids, transposons) vectors	Inefficient delivery to all/most cells of patients
			Limited maintenance and expression of the delivered gene
Genome editing (GE)	Experimental/first clinical studies	Introducing specific changes in the cells of MPS patients using modern molecular tools, like CRISPR/Cas9 or others	Limited efficiency of introducing genetic changes in all/most cells of patients

## Data Availability

All data generated or analyzed during this study are included in this published article.

## Supplementary Materials

The following supporting information can be downloaded at https://www.mdpi.com/article/10.3390/biom15101448/s1: Figure S1: The standardized mean values for body height z-scores for boys with MPS in calendar age classes. Figure S2: The standardized mean values for body height z-scores for girls with MPS in calendar age classes.
