# Supplementary material for: Mucopolysaccharidoses—What Clinicians Need to Know: A Clinical, Biochemical, and Molecular Overview
Source: Biomolecules. 2025 Oct 12;15(10):1448. doi: 10.3390/biom15101448 (PMC12562998; doi:10.3390/biom15101448)
Supplement: Supplementary file 1 [file biomolecules-15-01448-s001.zip › biomolecules-3885415-supplementary.pdf]

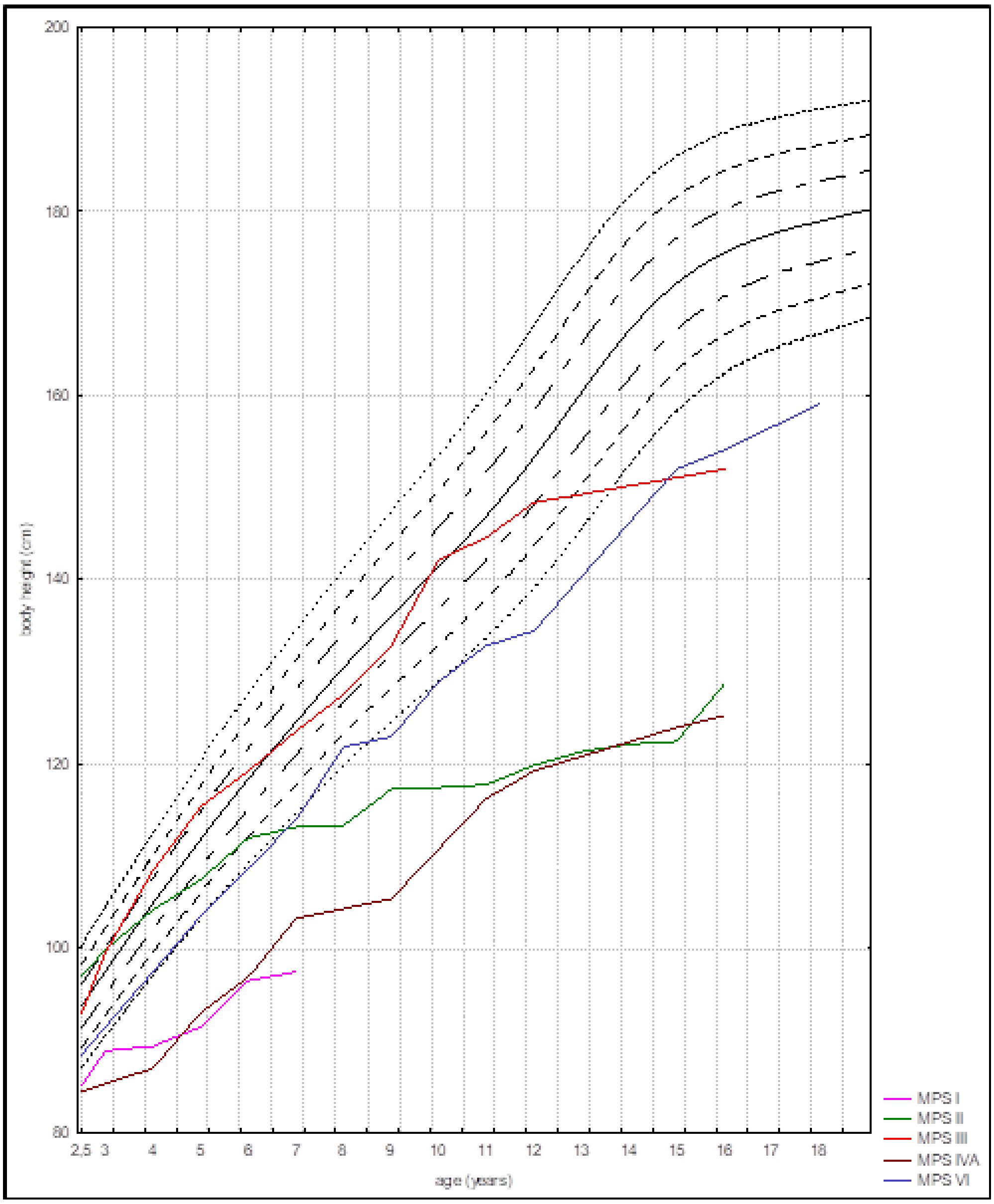

Figure S1. The standardized mean values for body height z-scores for boys with MPS in calendar age classes.

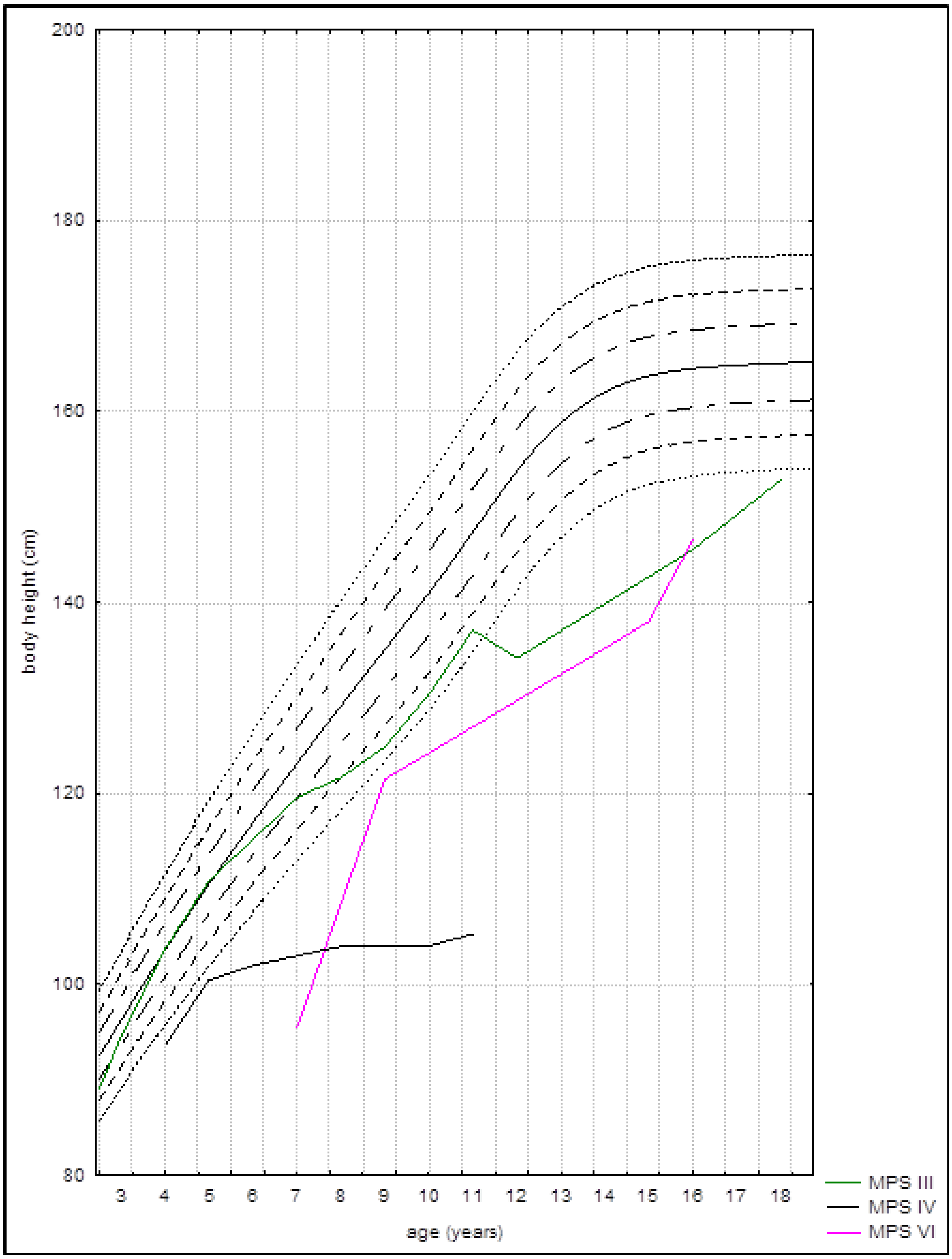

Figure S2. The standardized mean values for body height z-scores for girls with MPS in calendar age classes.
